# Fitness Cost of Daptomycin-Resistant *Staphylococcus aureus* Obtained from *in Vitro* Daptomycin Selection Pressure

**DOI:** 10.3389/fmicb.2017.02199

**Published:** 2017-11-09

**Authors:** Shuguang Li, Yuyao Yin, Hongbin Chen, Qi Wang, Xiaojuan Wang, Hui Wang

**Affiliations:** Department of Clinical Laboratory, Peking University People’s Hospital, Beijing, China

**Keywords:** *in vitro* resistance, mutation, daptomycin, *Staphylococcus aureus*, fitness cost

## Abstract

Daptomycin-resistant (DAP-R) *Staphylococcus aureus* strains are well documented, but have not been reported in China. To elucidate the evolution adaptability and fitness cost of DAP-R *S. aureus*, three DAP susceptible strains, Pre3 (MRSA, ST239-t037), Pre5 (MRSA, ST239-t037), and Pre14b (MSSA, ST188-t189), were isolated from patients with bloodstream infections, and serially passaged in Mueller–Hinton broth with a gradient of DAP concentration to select for resistance. Highly DAP-R mutants were obtained after screening for 34 passages. The DAP minimum inhibitory concentrations increased from 0.5 μg/ml in the parent strains to 16 μg/ml in the mutants, which remained tolerant to 4 μg/ml of DAP for more than 160 generations. The growth of the three mutant strains was slower than that of the parent strains, with relative fitness cost of 34.8%, 19.2%, and 15.0%, respectively. The *in vitro* serum tolerance of the mutants was decreased, and the lethality and pathogenicity in mice were weakened (*P* < 0.01). Transmission electron microscopy found that the cell walls of the mutants were significantly thicker (from 38.6% to 75.4%) than those of the parent cells. Mutation L826F of *mprF* was found in Post14b, G299V, and L473I of *mprF* and Y225N of *walK* were found in Post3, while T345A of *mprF*, S52N of *graS*, and F473I of *walK* were found in Post5. Thus, stable DAP-R mutants could be obtained from a middle-short term of *in vitro* DAP selection, and according to their fitness cost, some prevention and control work may be done to cope with DAP-R *S. aureus* that may appear in China in the future.

## Introduction

*Staphylococcus aureus* is a frequent cause of many hospital- and community-acquired infections of the skin and soft tissues, pneumonia, bacteremia, endocarditis, and sepsis, with substantial morbidity and mortality. The mortality of *S. aureus* bacteremia can be as high as 15–25% (Vogel et al., 2016). Infections due to *S. aureus* with heterogeneous intermediate resistance to vancomycin (hVISA/VISA) potentially fail vancomycin treatment, and daptomycin (DAP) is an important alternative treatment of methicillin-resistant *S. aureus* (MRSA) (Gould et al., 2013; Miller et al., 2016).

Daptomycin is a lipopeptide antibiotic produced by *Streptomyces roseosporus*, and was approved by the Food and Drug Administration (FDA) for soft-tissue infections in 2003 and for *S. aureus* bacteremia and right-sided endocarditis in 2006. DAP acts by calcium- and anionic phospholipid phosphatidylglycerol (PG)-mediated binding to the cell membrane, not the cell wall, of Gram-positive bacteria (Straus and Hancock, 2006). DAP exerts its bactericidal action against *S. aureus* in the stationary growth phase by inhibiting active metabolism without lysis of the target cell (Mascio et al., 2007; Cotroneo et al., 2008).

Daptomycin-resistant (DAP-R) *S. aureus* strains are well documented and of great concern when treating serious infections caused by this organism (Bayer et al., 2013; Miller et al., 2016). DAP resistance is associated with cell surface charge (*mprF*), regulation of *dltABCD* transcription, and global changes in the expression of VraSR and WalKR (YycFG), two genes that regulate cell membrane stress and maintenance (Bayer et al., 2013; Miller et al., 2016). Agr-defective mutants enhance *S. aureus* survival during DAP treatment (Pader et al., 2016). As is reported, *in vivo* DAP-R *S. aureus* arose during glycopeptide therapy, and the emergence of DAP-R isolates was preceded by a stable VISA or hVISA phenotype (Capone et al., 2016). However, *S. aureus* strains with this type of DAP resistance have not been reported in China. To further understand the drug resistance mechanism, and to prevent and cope with DAP-R *S. aureus* that may appear in China in the future, two MRSA strains (ST239-t037) and one methicillin-susceptible *S. aureus* (MSSA) (ST188-t189) from the major genotypes of bacteremia in China were selected (He et al., 2013), then highly DAP-R strains were generated *in vitro*, and their adaptations were studied.

## Materials and Methods

### Bacterial Strains

*Staphylococcus aureus* strains were isolated from hospitalized patients with bloodstream infections. These wild-type stains were named Pre3 (MRSA, ST239-t037), Pre5 (MRSA, ST239-t037), and Pre14b (MSSA, ST188-t189). All were sensitive to DAP, with minimum inhibitory concentrations (MICs) of 0.5 μg/ml.

### Experimental Selection of DAP-R *S. aureus*

Selection of DAP-R strains was conducted in 96-well microtiter plates with 200 μl Mueller–Hinton (MH) broth per well as previously described (Kim et al., 2014). The DAP gradient concentrations were 0, 0.25, 0.5, 1, 2, 4, 8, and 16 μg/ml with a calcium ion concentration of 50 μg/ml. The plates were incubated at 37°C for 20 h with agitation at 200 rpm. Culture supernatants with bacteria growing in the highest DAP concentrations were aspirated and continuously passaged in new DAP gradients until only DAP-R strains with MICs of not less than 8 μg/ml were obtained.

### Mutant Stability

The stability of the DAP-R-mutant strains was confirmed in serial cultures. After 24 h of culture in 20 ml Luria-Bertani (LB) broth at 35°C with agitation at 200 rpm, 200 μl of culture supernatant was removed; 100 μl was cultured in fresh LB broth, and 100 μl was plated on LB agar. After overnight culture, 100 colonies were randomly selected from the plates, and inoculated on MH agar plates with 4 μg/ml DAP and 50 μg/ml calcium ion in a 10 × 10 matrix (Wang et al., 2003; Dmowski et al., 2006).

### Growth Assay and Calculation of Generation Times

Wild-type and mutant strains were cultured overnight in LB broth, diluted to an OD_600_ of 0.01 and grown at 37°C with agitation at 200 rpm. The cell density was determined every 0.5 h by measuring the OD_600_. Both wild-type and mutant *S. aureus* strains were cultured to logarithmic growth phase (OD_600_ = 0.3) in LB broth at 37°C with agitation at 200 rpm. The number of colonies were counted at the beginning and after 1 h of time interval. Then the generation time was calculated as

(1) $$\begin{array}{c} G=\frac{t}{3.3\times \mathrm{lg}\frac{b}{B}}, \end{array}$$

where *G* = generation time, *t* = time interval, *b* = number of bacteria at the end of the time interval, and *B* = number of bacteria at the beginning of a time interval (Smits and Riemann, 1988).

### Drug Susceptibility Assay

The MICs of antibiotics in routine clinical use were determined in a central laboratory by broth microdilution following the CLSI M100-S26 guidelines (Clinical and Laboratory Standards Institute [CLSI], 2016).

### Fitness Measurements

DAP-sensitive (DAP-S) wild-type and the corresponding DAP-R strains were diluted to 0.5 × 10^7^ colony-forming units (CFU)/ml, equal volumes were combined, thus the initial ratio of the strain-pairs was infinitely close to 1:1, then 10 μl of the mixture was added to 20 ml LB broth and cultured at 35°C with agitation at 200 rpm. At 24-h intervals, 10 μl bacterial subcultures were transferred to fresh LB broth; meanwhile, 10 μl was inoculated on drug-free MH agar, and 10 μl on MH agar containing 1 μg/ml DAP. The number of DAP-S and DAP-R colonies were counted, and after 5 days, adaptive difference was calculated as

(2) $$\begin{array}{c} S=\ln\left[{\left(\frac{\frac{r_{t}}{s_{t}}}{\frac{r_{t-1}}{s_{t-1}}}\right)}^{\frac{1}{17}}\right], \end{array}$$

relative adaptive fitness as

(3) $$\begin{array}{c} F=1+S, \end{array}$$

and the fitness cost as

(4) $$\begin{array}{c} C=\left(1-F\right)\times 100\%, \end{array}$$

where *r*_t_ = number of resistant colonies and *s*_t_ = number of sensitive colonies (Sander et al., 2002; Guo et al., 2012).

### Serum Tolerance Assays

For the *in vitro* assay of serum tolerance, both wild-type and mutant *S. aureus* strains in logarithmic growth phase (OD_600_ = 0.3) were diluted to 2 × 10^6^ CFU/ml with 0.9% saline; 50 μl of the culture was mixed with 150 μl of serum from a healthy person in a 1.5 ml Eppendorf tube and cultured at 37°C with agitation at 200 rpm. At 0, 60, 120, and 180 min, 10 μl of the mixture was aspirated, combined with 90 μl MH broth, and plated on MH agar. Bacterial colonies were counted after overnight incubation; each strain was tested three times (Yeh et al., 2012). For the *in vivo* assay of serum tolerance, wild-type and the corresponding mutant *S. aureus* strains in logarithmic growth phase were diluted to 1 × 10^7^ CFU/ml, 50 μl of each culture was mixed and then inoculated into the tail vein of female Balb/c mice. After 72 h, the percentages of DAP-S and DAP-R bacteria in the blood and liver were measured. The animal experiments were approved by the research ethics board at Peking University People’s Hospital.

### Mouse Pathogenicity and Lethal Ability Assay

DAP-S and DAP-R strains and a *S. aureus* 8325-4 (a high-virulent strain) (Novick, 1967; Liu et al., 2010) control strain were incubated at 37°C with agitation at 200 rpm overnight and then adjusted to a concentration of 5 × 10^9^ CFU/ml with 1× PBS. Then 100 μl volumes of bacterial culture were injected into the tail veins of 15 female BALB/c mice at 8–10 weeks of age. Seven control mice were injected with 100 μl of 1× PBS. The baseline characteristics of the mice in each group did not differ. The mice were monitored for 15 days, every 3 h for the first 3 days and every 12 h for the next 12 days (Li et al., 2009).

### Phenotypic Analysis

Cell wall thickness was determined by transmission electron microscopy (FEI Tecnai Spirit). The fluorescence signals of cells were recorded with an EMCCD camera (Photometrics Evolve 512), and the signal intensity was used as a measure of cell membrane potential (Guo et al., 2015; Prindle et al., 2015).

### Sequencing of Suspected Genes Probably Associated with Daptomycin Resistance

Genomic DNA was isolated from both wild-type and mutant *S. aureus* strains using DNA Extraction Kit (TIANGEN Biotech, China). PCR amplification of *mprF, graRS, walKR, dltABCD*, and *agrABCD* was performed using the primers showed in Supplementary Table S1. The nucleotide sequence data are available at GenBank under the following accession numbers MG029205-MG029258 and MG242038-MG242061.

### Statistical Analysis

Statistical analysis of growth rate was performed with the software GraphPad Prism version 5 using one-way analysis of variance (ANOVA). Continuous variables were statistically analyzed with independent *t*-test. *P* < 0.05 was considered to be statistically significant.

## Results

### DAP-R *S. aureus* Mutants

Highly resistant Post14b, Post3, and Post5 mutants with MICs = 16 μg/ml were generated by continuous passage of susceptible *S. aureus* Pre14b, Pre3, and Pre5. Bacteria liquids after serial passage were collected and coated on LB agar separately, the morphology of the growing strains in a plate was quite the same, so we randomly picked single colony and did the following study. The sensitivity of *S. aureus* to DAP reduced gradually over time, and as shown in **Table 1**, after 34 days of serial passage, the DAP MICs increased to 16 μg/ml. The MICs of vancomycin and teicoplanin also increased; and those of linezolid, tedizolid, and ciprofloxacin decreased. Little change was observed in the MICs of the other tested antibiotics.

**Table 1:** Activity of daptomycin and other antibiotics against *S. aureus* wild-type and mutant strains.

Strains	pre14b	post14b	pre3	post3	pre5	post5
DAP	0.5	16	0.5	16	0.5	16
VAN	1	2	1	4	1	2
TEC	0.5	1	2	4	1	4
OXA	0.5	0.5	>256	256	>256	256
FOX	4	4	>256	256	>256	>256
LNZ	4	0.032	2	1	2	1
TZD	0.5	0.25	0.25	0.125	0.5	0.125
CIP	8	0.125	64	16	>64	64
LVX	0.25	0.25	8	8	32	32
ERY	0.5	0.5	>256	>256	>256	>256
CLI	64	0.06	>256	>256	>256	256
GEN	0.5	1	>256	>256	>256	>256
RIF	≤0.016	≤0.016	≤0.016	≤0.016	0.125	4
SXT	2	0.125	64	64	32	32

*DAP, daptomycin; VAN, vancomycin; TEC, teicoplanin; OXA, oxacillin; FOX, cefoxitin; LNZ, linezolid; TZD, tedizolid; CIP, ciprofloxacin; LVX, levofloxacin; ERY, erythromycin; CLI, clindamycin; GEN, gentamicin; RIF, rifampin; SXT, trimethoprim–sulfamethoxazole. Data are MIC (microgram/milliliter).*

### Growth Rates of DAP-R Strains

**FIGURE 1:**
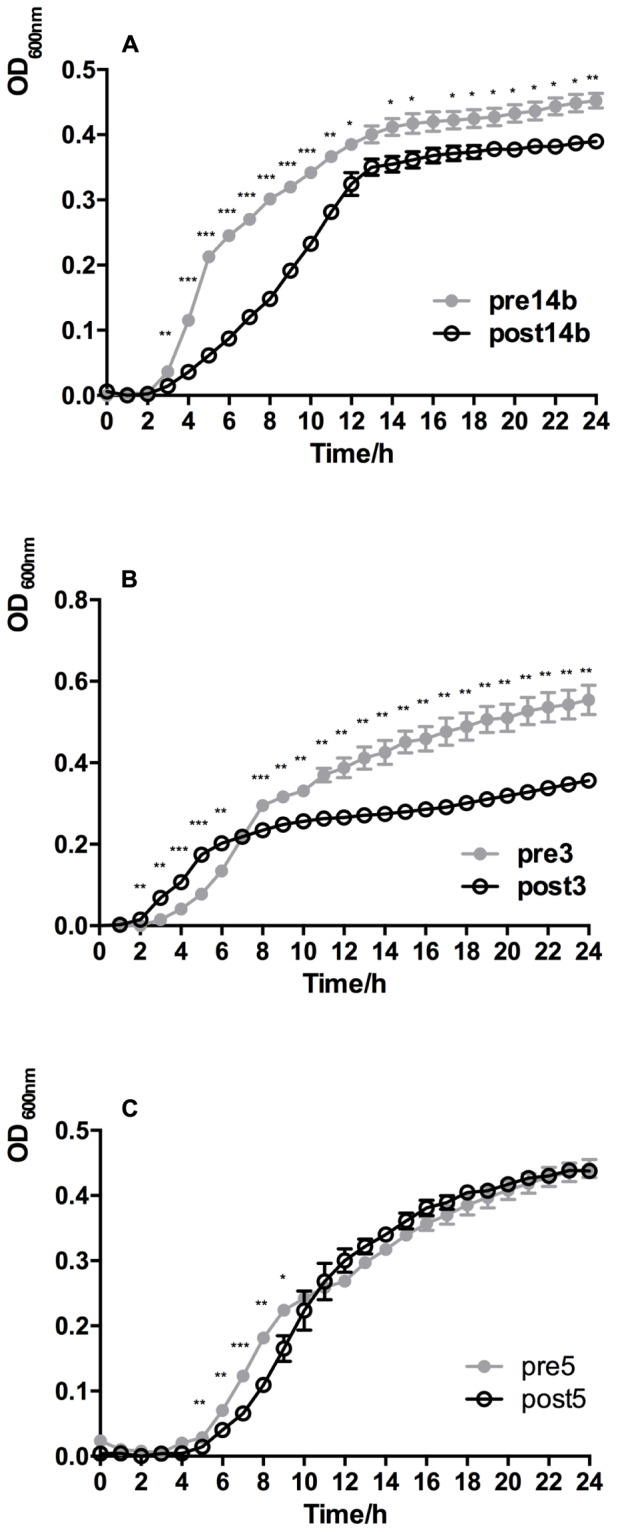
Growth characteristics of DAP-S *S. aureus* Pre14b, Pre3, and Pre5 strains and their corresponding isogenic derivative Post14b, Post3, and Post5 DAP-R mutants. **(A)** Pre14b and Post 14b, **(B)** Pre3 and Post3, and **(C)** Pre5 and Post5. Strain pairs were grown aerobically in LB liquid culture. The means and standard deviation of three independent experiments are shown. One-way ANOVA was used in statistical analysis. Symbols are defined in the insets. ^∗^*P* < 0.05, ^∗∗^*P* < 0.01, and ^∗∗∗^*P* < 0.001 for DAP-R vs. DAP-S by analysis of variance.

The growth rates of DAP-R mutant and DAP-S wild-type *S. aureus* strains are shown in **Figure 1**. The OD_600_ of the resistant Post14b strain was significantly lower than that of the Pre14b strain throughout the 24 h period of measurement (one-way ANOVA, *P* < 0.05). At each phase of the growth curve, the growth rates of the Post14b mutant were reduced compared with the Pre14b strain. The OD_600_ of the resistant Post3 strain was significantly higher than that of the Pre3 strain for the first 6 h, but then was lower from 8 to 24 h; considering the entire curve, growth of the Post3 strain was slower than that of the wild-type strain. The OD_600_ of the Post5 strain was significantly lower than that of the Pre5 strain from 5 to 9 h during logarithmic growth, but was not significantly different afterward, indicating similar growth rates.

### Stability of Resistance in Mutants

When grown on MH agar plates with 4 μg/ml DAP and 50 μg/ml calcium ion, DAP intolerant Post14b, Post3, and Post5 colonies were detected on days 7, 5, and 6, respectively, indicating stable resistance for 4–6 days of culture. Given generation times of 40 min for Post14b, 27 min for Post3b, and 45 min for Post5, resistance would be stable for 210 generations in Post14b and Post3b and 160 generations in Post5.

### Fitness Cost of Mutants

The relative fitness cost, or *C*-values were 34.8% for Post14b/Pre14b, 19.2% for Post3/Pre3, and 15.0% for Post5/Pre5. Thus, the isogenic derivative mutants all had an adaptive cost compared with the parent strains, with the Post14b mutant having the largest fitness cost (*C* = 34.8%).

### *In Vitro* and *in Vivo* Serum Tolerance of Mutants

The relative numbers of colonies of each strain counted in the *in vitro* assay of serum tolerance are shown in **Supplementary Figure S1**. The Post3 and Post14b strains had significantly lower serum tolerance than their wild-type counterparts. The Post5 strain had a lower tolerance for the first 2 h, but an increased tolerance in the third hour. In the assay of *in vivo* serum tolerance, the wild-type to the mutant strains were all >1000:1, with significant reductions in tolerance. The Pre3/Post3 ratios were 6212:1 in blood and 4174:1 in liver, respectively; the corresponding ratios were 121,266:1 and 1301:1 for Pre5/Post5 and 1192: 1 and 17,274:1 for Pre14b/Post14b in blood and liver, respectively.

### Pathogenicity and Lethality of Mutants

**FIGURE 2:**
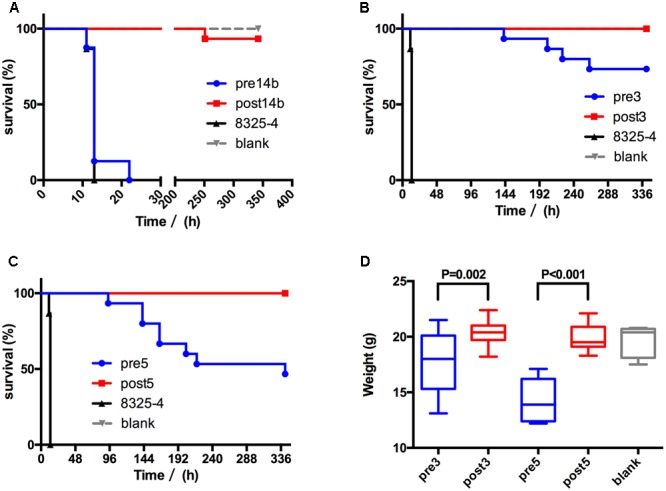
Pathogenicity and lethality ability of *S. aureus* in mice. The survival curves of **(A)** Pre14b/Post14b, **(B)** Pre3/Post3, and **(C)** Pre5/Post5 are shown. *S. aureus* 8325-4 was used as high toxicity control, and 1× PBS was used as blank control. **(D)** The body weight of surviving mice after infection with Pre3/Post3 and Pre5/Post5. Mice infected by 1× PBS were used as a blank control.

The survival and body weights of surviving mice were shown in **Figure 2**. The *S. aureus* 8325-4 control strain had the highest lethality; all 15 mice died within 12 h. All 15 mice infected with Pre14b died within 20 h, one mouse (7%) infected with Post14b died (**Figure 2A**), four (27%) infected with Pre3 and 8 (53%) infected with Pre5 died. No mice infected with Post3 or Post5 dies (**Figures 2B,C**). The body weights of surviving mice were 17.8 ± 2.6 g for Pre3, 20.3 ± 1.0 g for Post3, 14.3 ± 2.0 g for Pre5, and 20.3 ± 1.1 g for Post5. The mice infected with Pre3 and Pre5 were significantly lighter than those infected with their mutant strains (*P* < 0.05, **Figure 2D**). Both the pathogenicity and lethality of the resistant mutant strains were decreased.

### Cell Wall Thickness and Cell Membrane Potential

**FIGURE 3:**
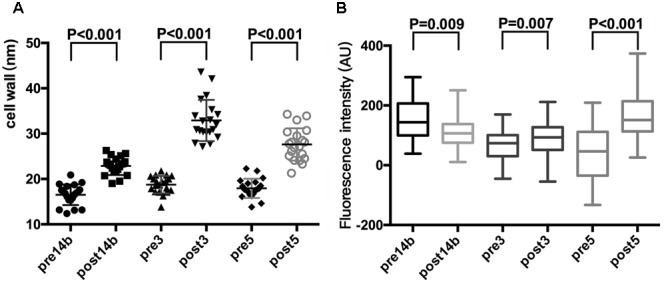
Cell wall thickness **(A)** and fluorescence intensity of the cell membrane **(B)** of *S. aureus* wild and mutant strains. Thickness and fluorescence intensity were statistically analyzed with independent *t*-test.

Cell wall thickness was increased and cell membrane potential was changed in the resistant strains compared with their sensitive counterparts. The cell wall thicknesses were 16.5 ± 2.3 nm in Pre14b and 22.9 ± 2.0 nm in Post14b, 18.8 ± 1.9 nm in Pre3 and 32.9 ± 4.5 nm Post3, and 17.9 ± 2.1 nm in Pre5 and 27.6 ± 3.6 nm in Post5. All increases in thickness were significant (*P* < 0.001), and were 38.6% in Pre-Post14b, 75.4% in Pre-Post3b, and 54.0% in Pre-Post5 (**Figure 3A**). The fluorescence intensities of the cell membranes were 153.0 ± 66.0 AU for Pre14b, 63.9 ± 49.9 AU for Pre3, and 41.8 ± 88.8 AU for Pre5. The corresponding values for the resistant strains were 109.8 ± 48.2 AU, 93.4 ± 56.2 AU, and 164.2 ± 78.7 AU, which were a reduction of 28.3% in Post14b and increases of 46.1% in Post3 and 292.5% in Post5. All the changes in fluorescence intensity indicated significant changes in cell membrane potentials in the mutant strains (*P* < 0.01, **Figure 3B**).

### Detection of Single-Nucleotide Polymorphisms in DAP-R Strains

The suspected genes may be related with DAP resistance were sequenced, single-nucleotide polymorphisms (SNPs) were found in *mprF, graS*, and *walK*, and the mutations of amino acid sequences were shown in **Table 2**. In detail, for Post14b, mutation was found in L826F of *mprF*; for Post3, mutation was found in G299V and L473I of *mprF*, Y225N of *walK*; for Post5, mutation was found in T345A of *mprF*, S52N of *graS*, and F473I of *walK*. However, no SNPs were found in *graR, walR, dltABCD*, and *agrABCD*.

**Table 2:** Amino acid changes in mutant *S. aureus* strains.

Strain pairs	*mprF*	*graS*	*walK*
Pre14b–Post14b	L826F	No SNP	No SNP
Pre3–Post3	G299V; L473I	No SNP	Y225N
Pre5–Post5	T345A	S52N	F473I

*Thirteen genes, *mprF, graR, graS, walK, walR, dltA, dltB, dltC, dltD, agrA, agrB, agrC*, and *agrD*, were sequenced, and mutation only detected in *mprF, graS*, and *walK*.*

## Discussion

The benefits of DAP for severe infections caused by multidrug-resistant Gram-positive pathogens include bacterial killing without lysing, reduced inflammatory reactions; rapid, concentration-dependent sterilization, a unique mechanism of action without cross-resistance with other antibiotics, and a bactericidal effect on bacterial growth and stability. DAP can also be used as a first-line treatment of life-threatening sepsis or in patients with renal dysfunction in cases with a history of glycopeptide use or a vancomycin MIC > 1.0 μg/ml (Gould et al., 2013; Gonzalez-Ruiz et al., 2016; Miller et al., 2016).

Daptomycin was approved by the American FDA in 2003 and the European EMA in 2006. Cases of clinical treatment failure because of DAP-R strains have been reported (Hsu et al., 2010; Twele et al., 2010), and the emergence of resistance may be associated with high bacterial loads and low doses of DAP (≤6 mg/kg/day) in severe infections such as endocarditis. In 2015 and 2016, the State FDA approved the production of DAP by three pharmaceutical companies in China. Consequently, DAP will probably become widely used in China. No clinical DAP-R *S. aureus* isolates have been reported in China, but highly resistant strains with MICs ≥ 16 μg/ml were obtained *in vitro* by a 34-day DAP induction. As the colonies grown from the passaged bacteria liquid have the same morphology, we presume that in a parallel test may be only one dominant colony could pass down under long time DAP selection; many parallel tests need to do at the same time to get variant resistant strains with different mutations. The MICs of two MRSA strains and the MSSA strain isolated from inpatients with bloodstream infections increased from 0.5 to 16 μg/ml and remained tolerant to 4 μg/ml DAP for more than 160 generations. The results confirmed that *S. aureus* could grow in the presence of high DAP concentrations, and that DAP resistance was stably inherited.

Relatively small increases in the MICs of vancomycin and teicoplanin were detected, no teicoplanin-resistant strains appeared, and only one mutant strain (Post3) had intermediate resistance to vancomycin (MIC = 4 μg/ml). The MIC increase of glycopeptides may be due to mutation in *mprF*, which changed in all three mutants and correlated with cell wall cross-linking. On the other hand, the MICs of linezolid, tedizolid, and ciprofloxacin decreased slightly. It was not determined whether that resulted in fitness cost or unknown genetic changes that may be revealed after genome sequencing. The MICs of oxacillin, cefoxitin, and the other tested antibiotics changed very little.

The growth rate of mutants was decreased significantly in two strain pairs (Pre14/Post14b and Pre3/Post3), and were similar in Pre5/Post5. The growth rate of the DAP-R mutants was not always slowed when cultured alone with sufficient medium. However, when cultured together with the wild-type strain and adequate nutrition *in vitro* or inoculated into mice *in vivo*, the number of mutant colonies was far less than the number of wild-type, and all mutant strains showed fitness cost with *C*-values between 15.0% and 34.8%. Because of fitness costs, the mutant strains performed weakly in the competitive growth experiments. *In vivo*, the conditions were more complex than *in vitro*, the decreased growth of the mutant strains may have resulted from both low competitiveness and low serum tolerance. The reductions of lethality and pathogenicity following infection in mice further confirmed the fitness costs of the DAP-R strains.

The fitness costs demonstrated by *C*-values were associated with changes in cell wall thickness and cell membrane potential. The cell wall thicknesses of all the mutant strains were significantly increased (*P* < 0.001), which is consistent with previous reports of vancomycin non-susceptible strains (Hanaki et al., 1998; Utaida et al., 2003). However, DAP and vancomycin resistance may be caused by different mechanisms, as the bacteria with thickened cell walls may be resistant to only one of the two drugs, such as Post14b and Post5, which had high DAP resistance, but both were sensitive to vancomycin (MIC ≤ 2 μg/ml).

*Staphylococcus aureus* cell membrane lipids are mainly negatively charged phospholipids such as PG and cardiolipin. When calcium ions are present, DAP causes cell death by binding to and depolarizing the cell membrane (Stefani et al., 2015). When changes in the composition of the cell membrane result in a decrease in its negative charge and increase in mobility, the resulting decrease in DAP binding leads to resistance. The decrease in cell membrane potential in the Post14b strain was consistent with this cause of DAP resistance. However, Post3 and Post5 had increased membrane potentials, which might not have favored DAP resistance; other factors that can lead to DAP resistance need to be evaluated.

In this study, all the DAP-R strains had a fitness cost that differed in each pair of DAP-R and DAP-S strains. Genetic mutations were found in *mprF, graS*, and *walK*. To our knowledge, G299V and L473I in *mprF*, S52N in *graS*, and Y225N and F473I in *walK* were the novel variations have not been reported. As *mprF* is associated with cell surface charge and cell wall cross-linking, WalK regulates cell membrane stress and maintenance, GraS regulates VraFG expression, involved in cationic antimicrobial peptide (CAMP) sensing and signal transduction to promote *S. aureus* resistance (Falord et al., 2012; Bayer et al., 2013; Miller et al., 2016), we presume that genetic changes of these three genes played important roles in DAP resistance. However, as drug resistance is complex property that depends on multigene activity, and the mutant strains not always showed consistent characteristics, other genes beside *mprF, graS*, and *walK* may be changed in the passages, thus whole-genome sequencing and comparative genome analysis of wild-type, final mutants, and resistant intermediates obtained during serial passage is warranted. What’s more, knockout and complementation strains of key genes would be constructed to confirm their roles in DAP resistance.

## Ethics Statement

The animal experiments were approved by the research ethics board at Peking University People’s Hospital, with the ERP No. 81371856 and IACUC Ethics Approval No. 2013-79.

## Author Contributions

HW conceived and designed the study. SL, YY, HC, QW, and XW performed experiments described in this study. SL and YY wrote the draft, and HW revised it. All authors approved the final version.

## Conflict of Interest Statement

The authors declare that the research was conducted in the absence of any commercial or financial relationships that could be construed as a potential conflict of interest.

## Supplementary Material

FIGURE S1 *In vitro* serum tolerance of wild-type and mutant strains.
