# Supplementary figures and images for: Fitness Cost of Daptomycin-Resistant Staphylococcus aureus Obtained from in Vitro Daptomycin Selection Pressure
Source: Front Microbiol. 2017 Nov 9;8:2199. doi: 10.3389/fmicb.2017.02199 (PMC5684181; doi:10.3389/fmicb.2017.02199)

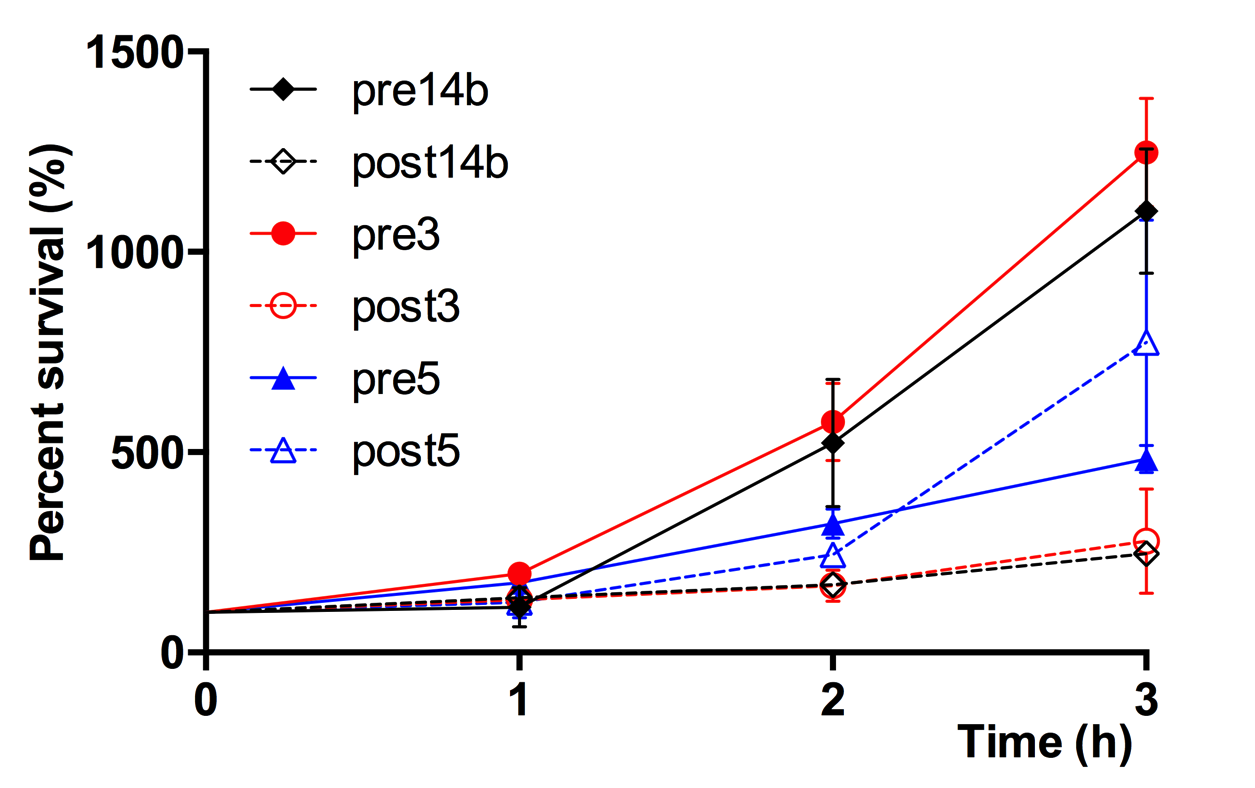

Supplement: FIGURE S1 — In vitro serum tolerance of wild-type and mutant strains. [file Image_1.TIFF]
