# Supplementary material for: Fitness Cost of Daptomycin-Resistant Staphylococcus aureus Obtained from in Vitro Daptomycin Selection Pressure
Source: Front Microbiol. 2017 Nov 9;8:2199. doi: 10.3389/fmicb.2017.02199 (PMC5684181; doi:10.3389/fmicb.2017.02199)
Supplement: Supplementary file 2 [file Table_1.docx]

**Table S1** Prime pairs of suspected genes may be involved in daptomycin resistance

| Gene | upstream primer | downstream primer |
| --- | --- | --- |
| *mprF* | ATGAATCAGGAAGTTAAAAAC | TTATTTGTGACGTATTACACG |
| *graR* | ATGCAAATACTACTAGTAGAAG | TTATTCATGTGCCATATATCC |
| *graS* | ATGAATAATTTGAAATGGG | TTAAAATGACAAATTTGTCAC |
| *walK* | ATGAAGTGGCTAAAACAAC | TTATTCATCCCAATCACC |
| *walR* | ATGGCTAGAAAAGTTGTTG | CTACTCATGTTGTTGGAGG |
| *dltA* | ATGACAGATATTATTAACAAGCTG | TCATCCGTTAATTACCTCTG |
| *dltB* | ATGATTCCATATGGTGATTT | TTATATAAGTTTACCTGAGAAGATT |
| *dltC* | ATGGAATTTAGAGAACAAGTATT | TCATCGTAACTCTTCTAATGC |
| *dltD* | ATGAAATTAAAACCTTTTTTAC | TTAATTTTTAGGTTTATCTACTTC |
| *agrABCD* | CTGCAGCAACTAAAAAGAAGC | CAGCTGGATCATCTTTAAAAAT |
